# Supplementary material for: Bias in control selection associated with the use of rapid tests in influenza vaccine effectiveness studies
Source: medRxiv. 2024 Nov 18:2024.11.16.24317422. Preprint. [Version 1] doi: 10.1101/2024.11.16.24317422 (PMC11601741; doi:10.1101/2024.11.16.24317422)

Bias in control selection associated with the use of rapid tests in influenza vaccine effectiveness studies

Eero Poukka, Caitriona Murphy, Loretta Mak, Samuel M. S. Cheng, Malik Peiris, Tim K. Tsang, Sheena G. Sullivan, Benjamin J. Cowling

Contents

Table S1. Detailed rapid test specification. 2

Table S2. Characteristics of participants according to rapid test of influenza A. 3

Table S3. Characteristics of participants according to rapid test of influenza B. 4

Table S4. Vaccine effectiveness (VE) against influenza A among participants aged less than 18 years (≥18 years) based on PCR or rapid test with 95% confidence interval 5

Table S5. Vaccine effectiveness (VE) against influenza A among participants aged 18 years or more (18 years) based on PCR or rapid test with 95% confidence interval 6

Figure S1. Study enrolment. 9

Figure S2. Influenza A and B cases over calendar time 9

Table S1. Detailed rapid test specification.

Assay	SARS-CoV-2 & Influenza A/B & RSV Antigen Kit (Colloidal Gold)
Manufacturer	Goldsite Diagnostics Inc., Shenzhen, China
Country of Origin	P.R. China
Certification	CE-IVD
Swab Type	Nasal swab
Reported Sensitivity and specificity	SARS-CoV-2: 93.0%; 100%<br>Influenza A: 85.0%; 99.3%<br>Influenza B: 95.0%; 100%<br>RSV: 92.3%; 100%
Format	Cassette
Method	Colloidal Gold
Volume applied into card/cassette	2 drops (~60µL)
Incubation	15 minutes
Readout	Visual: colored band
Limit of detection	SARS-CoV-2: 49 TCID 50/mL Influenza A(H3N2): 4.0 x 104 TCID 50/mL<br>Influenza A(H1N1): 2.0 x 104 TCID 50/mL RSV: 1.6x10^4 TCID 50/mL
Cross reactivity against other human virus	The results showed no cross reactivity

Table S2. Characteristics of participants according to rapid test of influenza A.

	False negative	False positive	True negative	True positive
N	97	5	1,276	313
Sex				
Male	30	2	571	149
Female	67	3	705	164
Age groups				
0–4	5	1	231	54
5–9	9	1	288	54
10–17	13	0	177	46
18–49	47	3	410	108
50+	23	0	170	51
Comorbidities				
Yes	11	0	118	25
No	86	5	1,158	288
Influenza vaccination for season 2023/24				
Yes	22	1	496	77
No	75	4	807	236
Previous season influenza vaccination (2022/23)				
Yes	24	0	421	77
No	73	5	855	236
Number of COVID-19 vaccinations				
0	10	1	252	70
1–2	25	0	354	73
≥3	62	4	670	170
Days since symptom onset				
One days	60	3	681	188
Two days	9	0	201	57
Three days	28	2	394	68

Table S3. Characteristics of participants according to rapid test of influenza B.

	False negative	False positive	True negative	True positive
N	36	1	1,512	142
Sex				
Male	22	0	663	67
Female	14	1	849	75
Age groups				
0–4	2	0	274	15
5–9	6	1	326	19
10–17	5	0	195	36
18–49	21	0	482	65
50+	2	0	235	7
Presence of chronic medical conditions				
Yes	3	0	143	8
No	33	1	1,369	134
Influenza vaccination for season 2023/24				
Yes	1	1	544	23
No	35	0	968	119
Previous season influenza vaccination (2022/23)				
Yes	3	1	495	23
No	33	0	1,017	119
Number of COVID-19 vaccinations				
0	4	0	313	16
1–2	12	0	399	41
≥3	20	1	800	85
Days since symptom onset				
One day	17	0	852	63
Two days	6	1	234	26
Three days	13	0	426	53

Table S4. Vaccine effectiveness (VE) against influenza A among participants aged less than 18 years by PCR or rapid test with 95% confidence interval.

	**VE by PCR (95% CI)**	**VE by rapid test (95% CI)**	**Bias-corrected VE by rapid test (95% CI)***	**Bias-corrected VE by rapid test (95% CI)†**
1. Exclusion of influenza B and SARS-CoV-2 by PCR (N=738) ‡	55% (30–72%)	53% (24–71%)	55% (33–70%)	55% (33–70%)
2. Exclusion of influenza B by PCR and adjusting for COVID-19 vaccination (N=796) ‡	52% (25–70%)	50% (20–69%)	52% (41–62%)	53% (42–62%)
3. Exclusion of influenza B by PCR (N=796)	52% (25–70%)	51% (21–70%)	53% (8–76%)	54% (31–69%)
4. All study participants (N=879)	47% (17–67%)	46% (13–67%)	48% (14–69%)	48% (22–65%)
5. Exclusion of influenza B and SARS-CoV-2 by rapid test (N=755)	53% (27–70%)	50% (21–69%)	53% (30–68%)	53% (30–69%)
6. Exclusion of influenza B by rapid test and adjusting for COVID-19 vaccination (N=808)	50% (48–86%)	48% (17–68%)	50% (39–59%)	51% (40–60%)
7. Exclusion of influenza B by rapid test (N=808)	50% (22–68%)	49% (18–68%)	51% (27–67%)	51% (27–68%)

VE = Vaccine effectiveness

* Diagnostic accuracy estimates based on data from the study participants (sensitivity = 85.1% and specificity = 99.7%)

† Diagnostic accuracy estimates of manufacturer (sensitivity = 85.0% and specificity = 99.3%)

‡ These estimates should be considered the most valid in this table.

Table S5. Vaccine effectiveness (VE) against influenza A among participants aged 18 years or more by PCR or rapid test with 95% confidence interval.

	VE by PCR (95% CI)	VE by rapid test (95% CI)	Bias-corrected VE by rapid test (95% CI)*	Bias-corrected VE by rapid test (95% CI)†
1. Exclusion of influenza B and SARS-CoV-2 by PCR (N=576) ‡	38% (-21–69%)	26% (-53–65%)	31% (-17–69%)	28% (-16–55%)
2. Exclusion of influenza B by PCR and adjusting for COVID-19 vaccination (N=717) ‡	41% (-10–69%)	32% (-35–67%)	37% (5–58%)	35% (12–53%)
3. Exclusion of influenza B by PCR (N=717)	41% (-9–69%)	31% (-38–66%)	36% (-128–82%)	34% (-4–58%)
4. All study participants (N=812)	37% (-19–67%)	26% (-50–65%)	29% (-51–66%)	28% (-12–54%)
5. Exclusion of influenza B and SARS-CoV-2 by rapid test (N=615)	37% (-21–68%)	26% (-52–65%)	31% (-19–60%)	27% (- 17–55%)
6. Exclusion of influenza B by rapid test and adjusting for COVID-19 vaccination (N=740)	39% (-14–68%)	30% (-40–66%)	34% (2–56%)	33% (7–51%)
7. Exclusion of influenza B by rapid test (N=740)	39% (-14–68%)	29% (-43–65%)	34% (-11–60%)	31% (-8–56%)

VE = Vaccine effectiveness

* Diagnostic accuracy estimates based on data from the study participants (sensitivity = 69.4% and specificity = 99.5%)

† Diagnostic accuracy estimates of manufacturer (sensitivity = 85.0% and specificity = 99.3%)

‡ These estimates should be considered the most valid in this table.

Table S6. Vaccine effectiveness (VE) against influenza A by PCR or rapid test with 95% confidence interval during H3N2 predominance (December 2023 – February 2024).

	**VE by PCR (95% CI)**	**VE by rapid test (95% CI)**	**Bias-corrected VE by rapid test (95% CI)***	**Bias-corrected VE by rapid test (95% CI)†**
1. Exclusion of influenza B and SARS-CoV-2 by PCR (N=751) ‡	59% (31–76%)	54% (20–77%)	58% (35–73%)	57% (35–72%)
2. Exclusion of influenza B by PCR and adjusting for COVID-19 vaccination (N=871) ‡	56% (27–74%)	52% (18–73%)	57% (43–67%)	55% (42–66%)
3. Exclusion of influenza B by PCR (N=871)	58% (29–75%)	53% (19–73%)	57% (18–77%)	56% (34–71%)
4. All study participants (N=920)	56% (27–74%)	52% (17–73%)	55% (19–75%)	54% (31–70%)
5. Exclusion of influenza B and SARS-CoV-2 by rapid test (N=778)	58% (30–76%)	53% (19–74%)	57% (34–72%)	57% (34–72%)
6. Exclusion of influenza B by rapid test and adjusting for COVID-19 vaccination (N=882)	55% (26–74%)	51% (16–72%)	55% (40–66%)	54% (41–64%)
7. Exclusion of influenza B by rapid test (N=882)	57% (13–73%)	52% (18–73%)	56% (33–71%)	55% (33–70%)

VE = Vaccine effectiveness

* Diagnostic accuracy estimates based on data from the study participants (sensitivity = 76.3% and specificity = 99.6%)

† Diagnostic accuracy estimates of manufacturer (sensitivity = 85.0% and specificity = 99.3%)

‡ These estimates should be considered the most valid in this table.

Table S7. Vaccine effectiveness (VE) against influenza A by PCR or rapid test with 95% confidence interval during H1N1 predominance (March – August 2024).

	**VE by PCR (95% CI)**	**VE by rapid test (95% CI)**	**Bias-corrected VE by rapid test (95% CI)***	**Bias-corrected VE by rapid test (95% CI)†**
1. Exclusion of influenza B and SARS-CoV-2 by PCR (N=563) ‡	33% (-14–61%)	30% (-25–61%)	33% (-8–59%)	32% (-8–57%)
2. Exclusion of influenza B by PCR and adjusting for COVID-19 vaccination (N=642) ‡	34% (-10–61%)	32% (-21–62%)	35% (11–52%)	33% (12–48%)
3. Exclusion of influenza B by PCR (N=642)	33% (-12–61%)	29% (-25–61%)	33% (-24–64%)	32% (-8–57%)
4. All study participants (N=771)	21% (-34–54%)	17% (-48–54%)	19% (-49–56%)	18% (-26–47%)
5. Exclusion of influenza B and SARS-CoV-2 by rapid test (N=)	29% (-20–58%)	26% (-32–59%)	29% (-15–56%)	27% (-16–54%)
6. Exclusion of influenza B by rapid test and adjusting for COVID-19 vaccination (N=666)	30% (-17–59%)	27% (-29–60%)	30% (5–48%)	29% (9–45%)
7. Exclusion of influenza B by rapid test (N=666)	29% (-20–58%)	25% (-32–58%)	28% (-15–54%)	27% (-13–53%)

VE = Vaccine effectiveness

* Diagnostic accuracy estimates based on data from the study participants (sensitivity = 76.3% and specificity = 99.6%)

† Diagnostic accuracy estimates of manufacturer (sensitivity = 85.0% and specificity = 99.3%)

‡ These estimates should be considered the most valid in this table.

Figure S1. Study enrolment.

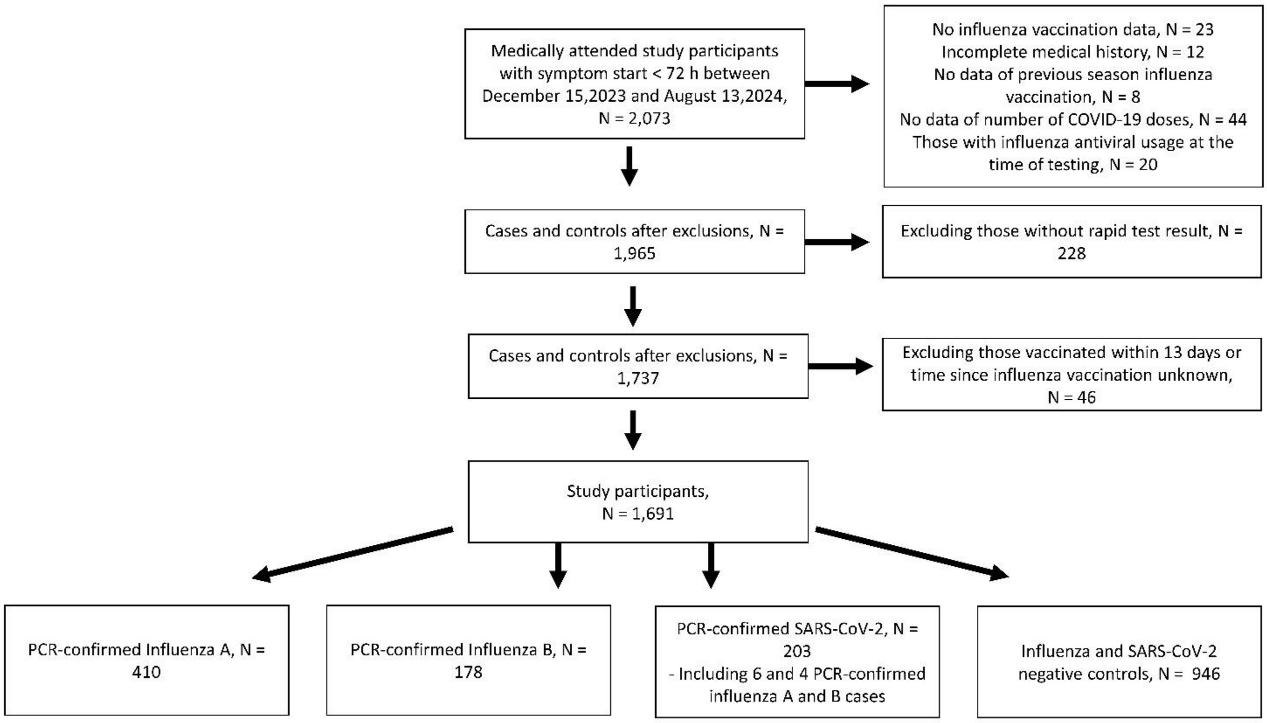

Figure S2. Influenza A and B cases over calendar time

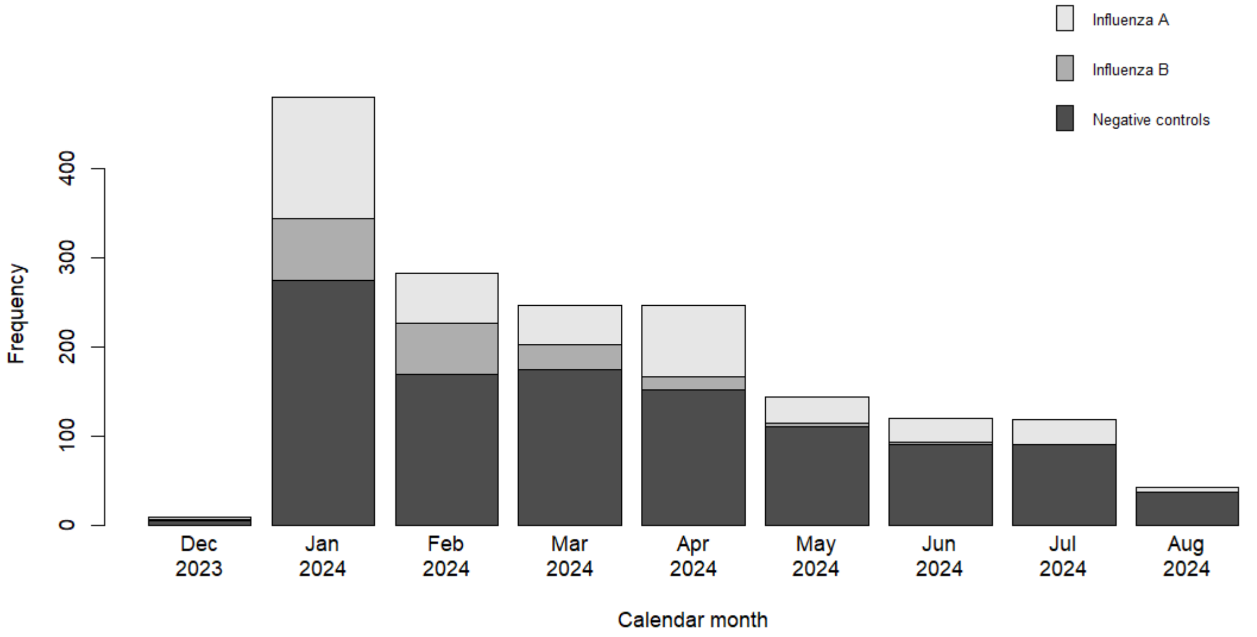

Supplement: Supplement 1 [file media-1.pdf]
